# Prospective study of Type 2 diabetes mellitus, anti‐diabetic drugs and risk of prostate cancer

**DOI:** 10.1002/ijc.30480

**Published:** 2016-11-03

**Authors:** Christel Häggström, Mieke Van Hemelrijck, Björn Zethelius, David Robinson, Birgitta Grundmark, Lars Holmberg, Soffia Gudbjörnsdottir, Hans Garmo, Pär Stattin

**Affiliations:** ^1^ Department of Surgical and Perioperative SciencesUrology and Andrology, Umeå UniversityUmeåSweden; ^2^ Department of Surgical SciencesUppsala UniversityUppsalaSweden; ^3^ Department of Biobank ResearchUmeå UniversityUmeåSweden; ^4^ Division of Cancer StudiesKing's College London, Faculty of Life Sciences and MedicineLondonUnited Kingdom; ^5^ Institute of Environmental MedicineKarolinska InstituteStockholmSweden; ^6^ Department of Public Health and Caring Sciences/GeriatricsUppsala UniversityUppsalaSweden; ^7^ Department of Scientific SupportMedical Products AgencyUppsalaSweden; ^8^ Department of Surgical SciencesRegional Cancer Centre Uppsala/ÖrebroUppsalaSweden; ^9^ Department of MedicineSahlgrenska University Hospital, Gothenburg UniversityGöteborgSweden

**Keywords:** prostate cancer, Type 2 diabetes mellitus, metformin, cohort study, survival analysis

## Abstract

Type 2 diabetes mellitus (T2DM) has consistently been associated with decreased risk of prostate cancer; however, if this decrease is related to the use of anti‐diabetic drugs is unknown. We prospectively studied men in the comparison cohort in the Prostate Cancer data Base Sweden 3.0, with data on T2DM, use of metformin, sulfonylurea and insulin retrieved from national health care registers and demographic databases. Cox proportional hazards regression models were used to compute hazard ratios (HR) and 95% confidence intervals (CI) of prostate cancer, adjusted for confounders. The study consisted of 612,846 men, mean age 72 years (standard deviation; SD = 9 years), out of whom 25,882 men were diagnosed with prostate cancer during follow up, mean time of 5 years (SD = 3 years). Men with more than 1 year's duration of T2DM had a decreased risk of prostate cancer compared to men without T2DM (HR = 0.85, 95% CI = 0.82–0.88) but among men with T2DM, those on metformin had no decrease (HR = 0.96, 95% CI = 0.77–1.19), whereas men on insulin (89%) or sulfonylurea (11%) had a decreased risk (HR = 0.73, 95% CI = 0.55–0.98), compared to men with T2DM not on anti‐diabetic drugs. Men with less than 1 year's duration of T2DM had no decrease in prostate cancer risk (HR = 1.11, 95% CI = 0.95–1.31). Our results gave no support to the hypothesis that metformin protects against prostate cancer as recently proposed. However, our data gave some support to an inverse association between T2DM severity and prostate cancer risk.

## Abstract 2

**What's new?**

Although Type 2 diabetes mellitus (T2DM) increases the risk of several cancers, multiple studies point toward a significantly inverse relationship between T2DM and prostate cancer risk in men. Use of the anti‐diabetic drug metformin is suspected of underlying the association. In this prospective study in Sweden, however, metformin failed to decrease the risk of prostate cancer. By comparison, risk was decreased in association with the use of insulin or sulfonylurea. These findings add some support to an inverse association between T2DM severity and prostate cancer risk.

**Abbreviations**
ATC: anatomical therapeutic chemical
BPH: benign prostatic hyperplasia
CCI: Charlson comorbidity index
CI: confidence interval
HR: hazard ratio
PCBaSe: prostate cancer data base Sweden
PSA: prostate‐specific antigen
SD: standard deviation
T1DM: Type 1 diabetes mellitus
T2DM: Type 2 diabetes mellitus

Type 2 diabetes mellitus (T2DM) has consistently been associated with decreased risk of prostate cancer^1^, ^2^, ^3^, ^4^, ^5^, ^6^ and recent data have suggested that the decrease in risk is related to the use of anti‐diabetic drugs.^7^ In particular metformin, currently one of the most commonly prescribed anti‐diabetic drugs, has been recently investigated with inconsistent results; some studies have found a decreased risk of prostate cancer among metformin users,^8^, ^9^, ^10^ while others have found no association.^11^, ^12^, ^13^, ^14^ However, several of these studies have not included data on diabetes mellitus type, duration, or severity, which is essential to distinguish the effect of the T2DM diagnosis *per se* from use of anti‐diabetic drugs. Furthermore, detailed data on the date of onset of T2DM, anti‐diabetic drug use and prostate cancer are necessary to investigate potential detection bias,^15^, ^16^ and other time‐related biases^17^, ^18^ that could influence the association.

The aim of study was to assess the risk of prostate cancer for men with T2DM in a large population‐based cohort study, taking duration of T2DM, use and duration of anti‐diabetic drugs, prostate cancer risk categories, comorbidity and socioeconomic status into account. To investigate the role of T2DM and possible detection bias related to diagnostic activity related to lower urinary tract symptoms, we also assessed the risk of benign prostatic hyperplasia for men with T2DM.

## Material and Methods

### Comparison cohort in the prostate cancer data base Sweden 3.0

Our study was designed as a prospective study in the comparison cohort in the Prostate Cancer data Base Sweden (PCBaSe) 3.0, which consists of men free from prostate cancer selected from the Swedish population.^19^ For each man with prostate cancer in the National Prostate Cancer Register (NPCR) of Sweden, five prostate cancer‐free men were selected into the comparison cohort, matched on birth year and county of residence. Using the Swedish 10‐digit personal identity number men with prostate cancer and prostate cancer‐free men were linked to other national health care registers and demographic databases.

We retrieved data from the National Patient Register on discharge diagnoses from hospital admissions up to 10 years prior to the date of inclusion in the comparison cohort and these data were used to calculate Charlson Comorbidity Index (CCI) as previously described.^20^, ^21^ CCI was categorized into four groups: CCI 0 = no comorbidity, and CCI 1, 2 and ≥3. Data on educational level were retrieved from the Longitudinal Integration Database for Health Insurance and Labor Market Studies at Statistics Sweden and categorized into three groups: ≤9 years, 10–12 years, and ≥13 years.^22^ Date and cause of death were obtained from the Cause of Death Register.

Start of study period was January 1, 2006, and end of study period was date of prostate cancer diagnosis, death, or December 31, 2013, whichever came first. To get a broader understanding of the association between T2DM and prostatic diseases, we also investigated the risk of a diagnosis of benign prostatic hyperplasia (BPH, International Classification of Diseases, 10th revision, code N409) obtained from the National Patient Register as a proxy for diagnostic activity related to lower urinary tract symptoms.

### Identification of men with T2DM

The National Diabetes Register of Sweden, initiated in 1996, has steadily increased its coverage and included 92% of the estimated number of prevalent cases of diabetes mellitus in 2013.^23^ Men registered in the National Diabetes Register have recorded details of diabetes mellitus including year of onset and type and were thus considered to have complete data on diabetes mellitus for the purpose of our study.^24^ Men with a registered diagnosis of T2DM (International Classification of Diseases, 10th revision, code E11.0–11.9) in the National Patient Register or in the Cause of Death Register, or men with at least two filled prescriptions of an anti‐diabetic drug in the Prescribed Drug Register were considered to have a diagnosis of diabetes mellitus, but with unknown onset year and type. We imputed data on onset year and type of diabetes for these men by use of multiple imputation based on predicted mean matching with five imputed datasets.^25^ Educational level, CCI, date of filled anti‐diabetic drug prescriptions and first registered date of T2DM diagnosis, or date of death due to T2DM, were used as independent variables for the imputation. After the imputation all men with diabetes mellitus were considered as complete cases with data on onset year and type. For men with T2DM, date of onset was set as July 1 of the registered or imputed onset year, first date of filled anti‐diabetic drug prescription or date of T2DM as registered in the National Patient Register, whichever event came first.

### Subgroup study of men with T2DM

To study risk of prostate cancer for men treated with anti‐diabetic drugs, we analyzed a subgroup of men with a T2DM onset after the start of the study period. This group of men had detailed information on anti‐diabetic drug prescriptions from the Prescribed Drug Register specifically on metformin (Anatomical Therapeutic Chemical; ATC code A10BA or A10BD), insulin (ATC code A10A) and sulfonylurea (ATC code A10BB). We categorized men with T2DM into three ordered groups: no anti‐diabetic drugs, metformin and insulin/sulfonylurea. These groups were based on the assumption that men who had filled a prescription for one or several anti‐diabetic drugs had continued to fill prescriptions for these drugs. Men in the metformin group had filled prescriptions metformin only, while men in the insulin/sulfonylurea group could also have filled prescriptions for metformin. The start date was defined as the date of the first filled prescription of each anti‐diabetic drug.

### Endpoints

Data on prostate cancer diagnosis were obtained from NPCR that includes information on date of diagnosis, age at diagnosis, tumor stage, Gleason score, serum levels of prostate‐specific antigen (PSA) at time of diagnosis and primary treatment.^19^ Prostate cancer risk categories were defined according to a modification of the National Comprehensive Cancer Network Guideline as previously described^26^: Low‐risk: T1‐2, Gleason score 2–6 and PSA < 10 ng/ml; intermediate‐risk: T1‐2, Gleason score 7 and/or PSA 10–20 ng/ml; high‐risk: T3 and/or Gleason score 8–10 and/or PSA 20–50 ng/ml; metastatic disease: T4 and/or N1 and/or PSA 50–100 ng/ml (regional metastases) or M1 and/or PSA > 100 ng/ml (distant metastases).^19^

### Statistical analysis

Risk of prostate cancer was calculated by use of hazard ratios (HR) in time‐updated Cox proportional hazard regression models with age as time‐scale, adjusted for educational level and CCI and stratified within the model for county of residence. Onset date of T2DM was used as a time‐updated covariate when evaluating the effect of T2DM on prostate cancer risk, whereas in the subgroup analysis of men with T2DM date of first prescription of metformin or insulin/sulfonylurea was used as a time‐updated covariate. The assumption of proportional hazards was tested with Schoenfeld residuals and found valid.

In the full study cohort, risk of prostate cancer was calculated for men with T2DM *versus* men without T2DM. To investigate a possible detection bias close to date of T2DM onset, we calculated risk of prostate cancer during the first year after T2DM onset, and more than 1 year after T2DM onset. Risk of prostate cancer in different risk categories was calculated similarly. We investigated the association between T2DM and BPH in a Cox model with the same adjustments and stratifications as described above.

In the subgroup of men with T2DM, risk of prostate cancer was calculated for men treated with metformin or insulin/sulfonylurea compared to men not treated with anti‐diabetic drugs. To distinguish between the association between duration of T2DM and anti‐diabetic drug use to prostate cancer risk, we calculated risk of prostate cancer during the first year and for more than 1 year after T2DM onset, and for less than or more than 1 year duration of anti‐diabetic drugs. Risk of prostate cancer classified into risk categories was analyzed similarly, but due to the limited size of the study population these risk categories were merged into two groups: favorable cancer that consisted of low‐risk and intermediate‐risk, and aggressive cancer that consisted of high‐risk and metastatic disease.

All analyses were performed with STATA MP/2 version 14.0 (StataCorp LP, College Station, TX). The study was approved by The Research Ethics Board at Umeå University, Sweden.

## Results

**Figure 1:**
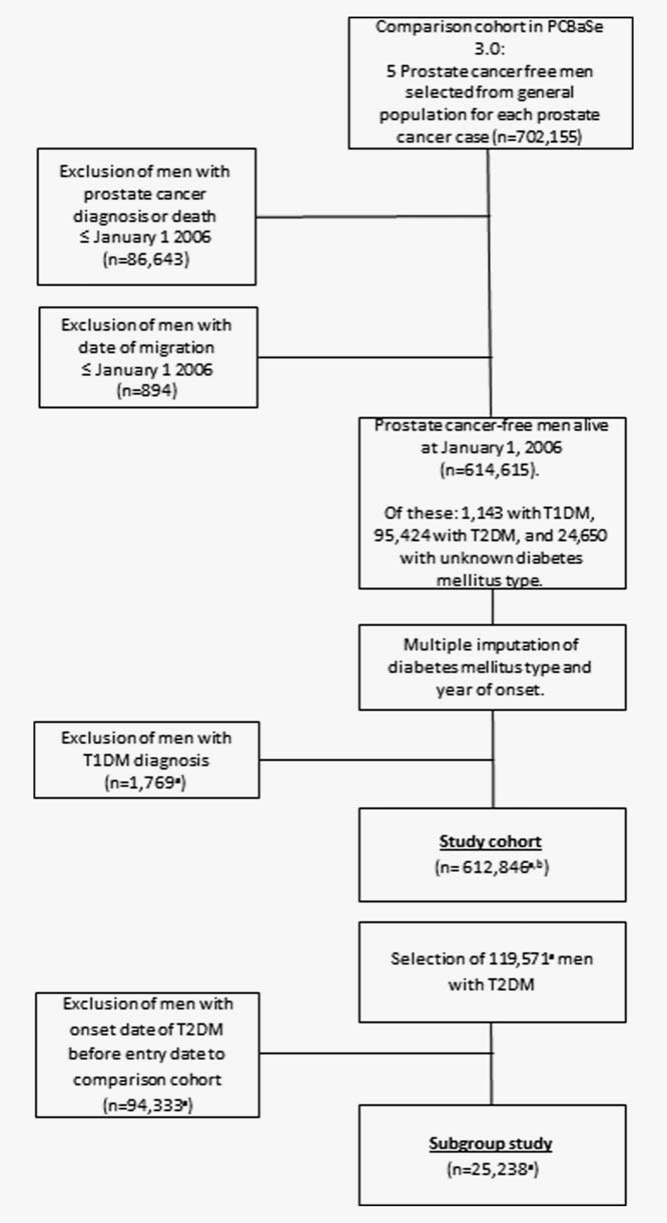
Selection of study population from the comparison cohort in the Prostate Cancer data Base Sweden 3.0^a^ Mean of five imputations^b^ Men with registered date of anti‐diabetic drug prescription (*n* = 2) or date of prostate cancer diagnosis (*n* = 1) after date of death were excluded.

**Figure 2:**
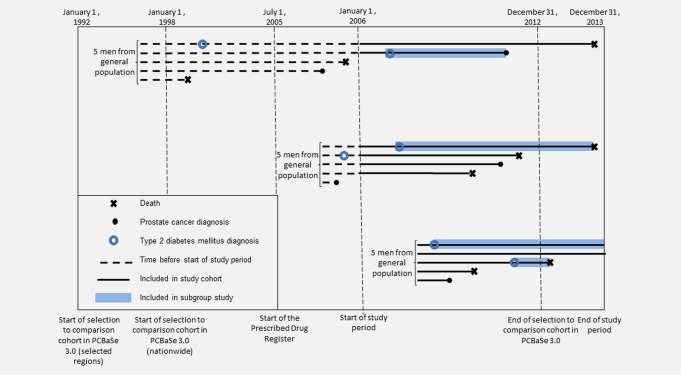
Timeline of recruitment of study population from the comparison cohort in the Prostate Cancer data Base Sweden 3.0. [Color figure can be viewed at wileyonlinelibrary.com]

**Figure 3:**
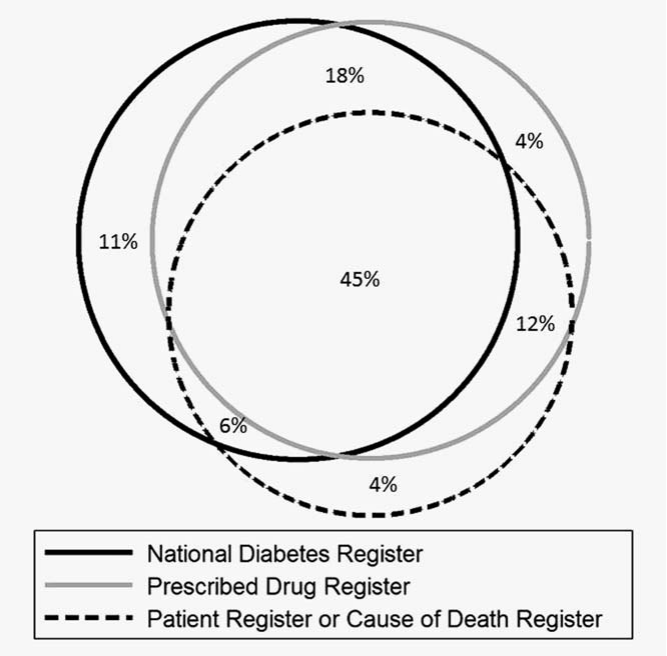
Proportion of men identified with Type 2 diabetes mellitus (T2DM) diagnosis in National Registers.

In the full study cohort of 612,846 men, a total of 119,571 men were diagnosed with T2DM during a mean follow‐up time of 5 years (SD = 3 years), 25,882 men were diagnosed with prostate cancer (Fig. 1 and Table 1). A timeline with dates used in the study is shown in Figure 2. Men with T2DM were identified in one or several of the registers as shown in Figure 3. Men with a duration of T2DM of more than 1 year had a decreased risk of prostate cancer compared to men without T2DM (HR = 0.85, 95% CI = 0.82–0.88) (Table 2). The risk was decreased for low‐risk, intermediate‐risk and metastatic prostate cancer (Supporting Information Table S1). Men with a duration of T2DM of less than 1 year had no decreased risk (HR = 1.11, 95% CI = 0.95–1.31). Risk of BPH was increased in men with a duration of T2DM of less than 1 year (HR = 1.39, 95% CI = 1.28–1.52), whereas men with more than 1 year duration had a smaller increase in risk of BPH (HR = 1.04, 95% CI = 1.01–1.06).

**Table 1:** Baseline characteristics of study population in Prostate Cancer data Base Sweden (PCBaSe), based on mean values of five imputations

	**Study cohort** a	**Subgroup of men with T2DM**
	***N* (%)**	***N* (%)**
Study population	612,846	25,238
Age at start of study		
≤59 years	60,552 (10)	1,273 (5)
60–64 years	91,557 (15)	3,221 (13)
65–69 years	115,846 (19)	5,385 (21)
70–74 years	109,449 (18)	5,592 (22)
75–79 years	99,901 (16)	4,935 (20)
≥80 years	135,541 (22)	4,836 (19)
Educational level^b^		
≤9 years	275,724 (45)	11,826 (47)
10–12 years	218,070 (36)	9,215 (37)
≥13 years	119,052 (19)	4,201 (17)
Charlson comorbity index		
No comorbidity	456,085 (74)	19,888 (79)
1	82,521 (13)	3,373 (13)
2	44,358 (7)	1,405 (6)
≥3	29,882 (5)	576 (2)
Mean years in follow up (SD)	5(3)	4(2)

a The maximum difference between the imputed dataset was 38 men.

b Educational level missing for 10,691 men (2%); these men were included in the group with ≤9 years.

Abbreviations: Standard deviation (SD).

**Table 2:** Hazard ratios (HR) and 95% confidence intervals of prostate cancer for men with Type 2 diabetes mellitus (T2DM)

	**Person‐years**	**# Cases**	**HR (95% CI)**
No T2DM	2,552,690	21,931	1.00 (ref)
T2DM	525,210	3,951	0.86 (0.83–0.90)
T2DM duration < 1 year	26,971	259	1.11 (0.95–1.31)
T2DM duration > 1 year	498,240	3,691	0.85 (0.82–0.88)

Cox regression models were based on time‐updated data of T2DM onset and adjusted for educational level, CCI and stratified for county. The hazard ratios are mean values based on regression models from five imputations.

In the subgroup of 25,238 men with T2DM and detailed information on anti‐diabetic drug use, 759 men were diagnosed with prostate cancer during a mean follow‐up time of 4 years (SD = 2 years). Compared to men not treated with anti‐diabetic drugs, men on metformin had no increased risk of prostate cancer (HR = 0.96, 95% CI = 0.77–1.19), whereas men on insulin (89%) or sulfonylurea (11%) had a decreased risk (HR = 0.73, 95% CI = 0.55–0.98) (Table 3). Men with a duration of T2DM of less than 1 year who were on metformin had an increased risk (HR = 1.49, 95% CI = 1.11–1.99), and in an analysis of prostate cancer risk categories, these men had a particularly increased risk of favorable cancer (HR = 1.76, 95% CI = 1.17–2.66) (Supporting Information Table S2A), whereas risk of aggressive cancer was not associated with any of the combinations of anti‐diabetic drugs or duration of T2DM (Supporting Information Table S2B).

**Table 3:** Hazard ratios (HR) and 95% confidence intervals of prostate cancer according to anti‐diabetic drug usage in the subgroup of men with Type 2 diabetes mellitus (T2DM)

	**T2DM duration < 1 year**			**T2DM duration > 1 year**		
	**Person‐years**	**# Cases**	**HR (95% CI)**	**Person‐years**	**# Cases**	**HR (95% CI)**
No anti‐diabetic drugs	15,410	148	1.21 (0.93–1.58)	29,802	257	1.00 (ref)
Metformin < 1 year	6,136	67	1.49 (1.11–1.99)	4,138	27	0.84 (0.55–1.27)
Insulin/sulfonylurea < 1 year	2,215	27	1.51 (0.99–2.30)	2,529	11	0.54 (0.29–0.99)
Metformin > 1 year	NA	NA	NA	20,558	157	0.96 (0.77–1.19)
Insulin/sulfonylurea >1 year	NA	NA	NA	10,320	65	0.73 (0.55–0.98)

Cox regression models were based on time‐updated data of anti‐diabetic drugs and adjusted for educational level, CCI and stratified for county. The hazard ratios are mean values based on regression models from five imputations.

## Discussion

In this large population‐based cohort study with comprehensive data on T2DM, anti‐diabetic drug use, prostate cancer risk categories and putative confounders, we found no decrease in risk of prostate cancer for men on metformin compared to men with T2DM not treated with anti‐diabetic drugs. In contrast, we found a decreased risk of prostate cancer for men on insulin or sulfonylurea.

Strengths of our study include access to several high‐quality, nationwide, population‐based registers with detailed data on T2DM, anti‐diabetic drug prescriptions, prostate cancer characteristics and confounding factors including comorbidity and socioeconomic factors.^19^, ^23^ We investigated several aspects of time‐related associations: age, time‐updated analysis of date of T2DM onset and anti‐diabetic drug use and duration of T2DM. Furthermore, duration of T2DM was analyzed to investigate detection bias or reverse causation.^15^, ^16^ To further assess if there was detection bias we used data on BPH as a proxy for diagnostic activity for lower urinary tract symptoms that can be present at the onset of diabetes. To distinguish between the association between T2DM and prostate cancer risk and anti‐diabetic drugs and prostate cancer risk, we investigated a subgroup of men with onset date of T2DM after the start date of the Prescribed Drug Register. Limitations of our study included a relatively short follow‐up time and a high mean age of the study population; however, there was a wide range in age from 50 to 101 years and we found no evidence that the association between T2DM and prostate cancer differed according to age. Other limitations of the study were the lack of data of risk factors of prostate cancer, for example, family history and body mass index, and this may have resulted in residual confounding.

Our overall risk estimates for prostate cancer for men with T2DM are in accordance with previous reports.^1^, ^4^, ^5^, ^27^, ^28^, ^29^, ^30^ In line with previous studies, shorter duration of T2DM was not associated with decreased risk of prostate cancer, whereas longer duration of T2DM was associated with a decreased risk.^1^, ^8^, ^15^, ^16^, ^17^, ^29^, ^31^, ^32^ For men with long T2DM duration, we found a decreased risk of both favorable and aggressive cancer, essentially in accordance with previous studies that have reported a decreased risk of localized prostate cancer^28^, ^30^, ^32^, ^33^ and all prostate cancer.^1^, ^34^, ^35^ Furthermore, men with T2DM in our study had an increased risk of BPH in accordance with previous studies,^36^, ^37^ and this risk was higher during the first year after T2DM onset, speculatively caused by detection bias due to investigation of urinary tract symptoms. To the best of our knowledge, no previous study has investigated risk of BPH taking duration of T2DM into account. We speculate that increase in diagnostic work‐up of lower urinary tract symptoms including PSA testing and subsequent biopsies, sometimes initiated due to glucosuria in men with newly diagnosed T2DM, will increase the probability of prostate cancer detection.^38^, ^39^

In the subgroup of men with T2DM with more than 1 year disease duration, we found no decrease in risk of prostate cancer for men on metformin and a weak decrease in risk for men on insulin/sulfonylurea compared to men with T2DM not on anti‐diabetic drugs. These results are in line with previous studies on metformin in a similar setting.^11^, ^40^

Other studies of men with T2DM have investigated the association between metformin and risk of prostate cancer with other referents than used in our study. One study investigated metformin *versus* sulfonylurea,^14^ one study compared ever‐users with never‐users of metformin^41^ and three meta‐analyses looked at metformin *versus* all other therapies combined.^42^, ^43^, ^44^ In brief, all these reports showed no difference between groups for overall prostate cancer risk. In addition, one study of insulin *versus* no insulin found a decreased risk of prostate cancer in insulin users, but no difference in risk when analyzing insulin *versus* other anti‐diabetic drugs;^13^ another study of insulin *versus* other glucose‐lowering agents found no difference in risk.^12^ In contrast, other observational studies found that men on metformin^7^, ^8^, ^10^ and insulin,^1^, ^7^, ^8^, ^31^ *versus* men not treated with these anti‐diabetic drugs, irrespective of T2DM status, had a decreased risk of prostate cancer.

Putative mechanisms for the decreased risk of prostate cancer for men with long duration of T2DM include low levels of androgens,^45^ a genetic profile with increased risk of T2DM and decreased risk of prostate cancer,^46^ high all‐cause mortality and low PSA levels but large prostate thereby decreasing the probability of biopsy and detection of small indolent cancers.^47^, ^48^ Our results can be generalized to populations with similar prostate cancer incidences and background mortality, that is, westernized countries.

In Sweden, metformin is the drug of choice for men with incident T2DM in need of pharmacotherapy, and with disease progression other anti‐diabetic drugs may be added at a later stage. Thus, a majority of men with a newly diagnosed T2DM will be prescribed metformin, as shown in our study. In line with this, the National Diabetes Register reported that men on insulin had a longer duration of T2DM and higher levels of glycated hemoglobin compared to men on metformin^49^ and men not on anti‐diabetic drugs. High levels of glycated hemoglobin and also insulin resistance have been associated with decreased prostate cancer risk suggesting that severe metabolic aberrations associated with poor T2DM control are associated with a decreased risk of prostate cancer.^50^

In conclusion, men with T2DM had a decreased risk of prostate cancer and but there was no association between metformin use and risk as previously proposed. More data are needed to elucidate if there is an inverse association between T2DM severity and prostate cancer risk.

## Supporting information

Supporting Information
