# Supplementary material for: Prospective study of Type 2 diabetes mellitus, anti‐diabetic drugs and risk of prostate cancer
Source: Int J Cancer. 2016 Nov 3;140(3):611–7. doi: 10.1002/ijc.30480 (PMC5215657; doi:10.1002/ijc.30480)
Supplement: Supplementary file 1 — Supporting Information [file IJC-140-611-s001.docx]

**Table 1.** Hazard ratios (HR) and 95% confidence intervals of prostate cancer in risk categories for men with type 2 diabetes mellitus (T2DM).

Cox regression models were based on time-updated data of T2DM onset and adjusted for educational level, CCI and stratified for county. The hazard ratios are mean values based on regression models from five imputations. Prostate cancer risk category information missing for 767 prostate cancer cases in the full cohort.

|  | **# cases** | **HR (95% CI)** |
| --- | --- | --- |
| **No T2DM** |  | 1.00 (ref) |
| **T2DM duration <1 year** |  |  |
| Low-risk | 41 | 0.82 (0.58 - 1.15) |
| Intermediate-risk | 74 | 1.13 (0.87 - 1.47) |
| High-risk | 76 | 1.24 (0.95 - 1.62) |
| Metastatic disease | 62 | 1.22 (0.88 - 1.68) |
| **T2DM duration >1 year** |  |  |
| Low-risk | 554 | 0.73 (0.66 - 0.80) |
| Intermediate-risk | 885 | 0.79 (0.74 - 0.85) |
| High-risk | 1,166 | 0.95 (0.89 - 1.02) |
| Metastatic disease | 965 | 0.88 (0.82 - 0.95) |

**Table 2.** Hazard ratios (HR) and 95% confidence intervals of a) low and intermediate-risk prostate cancer and b) high-risk and metastatic prostate cancer according to anti-diabetic drug usage in a subgroup of men with type 2 diabetes mellitus (T2DM).

Cox regression models were based on time-updated data of anti-diabetic drugs and adjusted for educational level, CCI and stratified for county. The hazard ratios are mean values based on regression models from five imputations.

**A**

|  | T2DM duration < 1 year | T2DM duration > 1 year |
| --- | --- | --- |
| No anti-diabetic drugs | 1.05 (0.72 - 1.54) | 1.00 (ref) |
| Metformin < 1 year | 1.76 (1.17 - 2.66) | 0.62 (0.30 - 1.27) |
| Insulin/sulphonylurea < 1 year | 1.29 (0.66 - 2.55) | 0.68 (0.30 - 1.62) |
| Metformin > 1 year | NA | 1.17 (0.85 - 1.60) |
| Insulin/sulphonylurea >1 year | NA | 0.66 (0.41 - 1.06) |

**B**

|  | T2DM duration < 1 year | T2DM duration > 1 year |
| --- | --- | --- |
| No anti-diabetic drugs | 1.35 (0.97 - 1.89) | 1.00 (ref) |
| Metformin < 1 year | 1.30 (0.85 - 1.98) | 1.08 (0.64 - 1.84) |
| Insulin/sulphonylurea < 1 year | 1.67 (0.98 - 2.85) | 0.46 (0.19 - 1.06) |
| Metformin > 1 year | NA | 0.77 (0.56 - 1.06) |
| Insulin/sulphonylurea >1 year | NA | 0.81 (0.55 - 1.18) |
